# Community mental health in India: A rethink

**DOI:** 10.1186/1752-4458-2-11

**Published:** 2008-07-14

**Authors:** Rangawsamy Thara, Ramachandran Padmavati, Jothy R Aynkran, Sujit John

**Affiliations:** 1 Schizophrenia Research Foundation, R/7A, North Main Road, Anna Nagar, West Extension, Chennai, 600101, India

## Abstract

**Background:**

Community care of the chronic mentally ill has always been prevalent in India, largely due to family involvement and unavailability of institutions. In the 80s, a few mental health clinics became operational in some parts of the country. The Schizophrenia Research Foundation (SCARF), an NGO in Chennai had established a community clinic in 1989 in Thiruporur, which was functional till 1999. During this period various programmes such as training of the primary health center staff, setting up a referral system, setting up of a Citizen's Group, and self-employment schemes were initiated. It was decided to begin a follow up in 2005 to determine the present status of the schemes as well as the current status of the patients registered at the clinic. This we believed would lead to pointers to help evolve future community based programmes.

**Methods:**

One hundred and eighty five patients with chronic mental illness were followed up and their present treatment status determined using a modified version of the Psychiatric and Personal History Schedule (PPHS). The resources created earlier were assessed and qualitative information was gathered during interviews with patient and families and other stakeholders to identify the reasons behind the sustenance or failure of these initiatives.

**Results:**

Of the 185 patients followed up, 15% had continued treatment, 35% had stopped treatment, 21% had died, 12% had wandered away from home and 17% were untraceable. Of the patients who had discontinued treatment 25% were asymptomatic while 75% were acutely psychotic.

The referral service was used by only 15% of the patients and mental health services provided by the PHC stopped within a year. The Citizen's group was functional for only a year and apart from chicken rearing, all other self-employment schemes were discontinued within a period of 6 months to 3 years.

There were multiple factors contributing to the failure, the primary reasons being the limited access and associated expenses entailed in seeking treatment, inadequate knowledge about the illness, lack of support from the family and community and continued dependence by the family on the service provider to provide solutions.

**Conclusion:**

Community based initiatives in the management of mental disorders however well intentioned will not be sustainable unless the family and the community are involved in the intervention program with support being provided regularly by mental health professionals.

## Background

The family and the community in India have always played a significant role in the management of the chronic mentally ill in the community [1]. Yet, there have been several community studies which have demonstrated that one third of the chronic mentally ill in the community remain untreated [2-4]. The need to "reach the untreated", given the inadequacies of mental health care facilities in India was the prime motivating factor for the community mental health movement.

As part of this effort, the Schizophrenia Research Foundation (SCARF, India), had started a community based mental health programme at Thiruporur in 1989 that was operational till 1999.

### Description of the original programme

#### Programme location

Tamil Nadu is one of the southern states of India. The Thiruporur block in the district of Chengalpattu is over 60 kms from Chennai, the capital city of Tamil Nadu. Spread over 140 sq.kms, its 102 villages have a total population of 150,000. The primary occupations are agriculture, fishing and salt making. In the last two decades, some small industries have sprung up here leading to better employment opportunities. Levels of education are low and most belong to the lower socio-economic class. Over 90% of the population are Hindus. One Primary Health Center (PHC), 4 sub-centers with six physicians, three rural dispensaries and six maternity centers provide general health and maternal health care to the population. The closest psychiatric facility is a state run hospital 22 kms away. There is no private psychiatric care.

In 1989, a Community based mental health programme was initiated here with funding support from the International Development Research Center, Canada. We decided to piggyback on an ongoing Community Based Rehabilitation (CBR) programme for the physically disabled by the Red Cross Society of India and trained 40 of their volunteers/community level workers.

#### The specific aims of the program initiated were

(1) To increase awareness about mental disorders and their treatments.

(2) To integrate a mental health component in the existing health care infrastructure by training primary care personnel (medical and paramedical) in dealing with mental health and psychosocial problems. It was believed that creation of this infrastructure of trained personnel would lead to the continued availability of mental health services and would enable our withdrawal without any major disruption in service delivery.

(3) To identify and develop community based rehabilitation strategies to be run by a network of trained lay volunteers. It was hoped that with the inclusion of this component an integrated mental health service model would be established. This would once again pave the way for our withdrawal as service providers.

#### Activities undertaken

##### Establishment of a mental health center

A mental health center was established in 1989 in the town of Thiruporur and was visited every fortnight by a team of mental health professionals, comprising of a psychiatrist and two psychiatric social workers. Medication, counseling and simple rehabilitation measures were offered. A study co-ordinator and three field investigators remained in the study area itself.

###### Training of primary care personnel

In 1994, as a parallel effort, the primary health care network in this area was also sensitized to detection of mental disorders. Eight physicians and 68 multi-purpose workers were trained over a period of three days using standardized manuals and audiovisual material. Case vignettes were also used. Measures were then taken to make available the required medication at the PHC

An active liaison was also established with the traditional healing centers in that area (one temple and one mosque frequented for healing of mental disorders). We also ensured that the process of traditional/religious healing was not interfered with, but persuaded the patient to accept the medicines also. This was accomplished in many cases with the help of volunteers and authorities at the places of worship.

A study on explanatory models of mental illness was undertaken [5]. It was evident that help seeking patterns of the population was to a large extent determined by their explanatory models.

###### Citizen's group

The CBR program also initiated the formation of a citizens' group, members of the village such as village leaders, schoolteachers, heads of religious institutions in the area and interested community persons. This group functioned to create awareness in the community, encourage help seeking, organize mental health camps in various villages, reduce stigma and involve the community in general in the rehabilitation of the patients. At a personal level, the members spoke to the families of patients registered at the center and ensured good follow-up. The members created a small fund, tapping fiscal resources amongst themselves, to enable activities related to mental health care provision.

#### Withdrawal from the area

From 1994 onwards, we began referring patients to the PHC. Initially, all persons suffering from seizure disorders, and minor mental morbidities were referred. Those who were acutely ill were referred to the nearest hospital, which had a psychiatry department. However, we found that many with psychoses continued to visit our center since the medicines at the PHCs seemed to be inadequate. The children with Mental Retardation (MR) were referred to the appropriate NGOs. We had to close our center in 1999 due to lack of funds. For the sake of continuity, we retained two of our staff in the study area till 2002 – the study co-ordinator and a field investigator. They would visit the homes of these patients to determine the current status. This team found that about 63% of the persons we had referred to other agencies were continuing treatment. It was after this information coupled by total lack of resources that we withdrew totally from the area in 2002.

When we visited the area in 2005 in connection with another study we decided to have a relook at the current situation.

### Present study

At the time of our exit from Thiruporur we had about 440 patients attending the clinic regularly.

The diagnostic break up was as below:

1. Psychotic disorders = 185

2. Minor mental morbidity (anxiety and depression) = 70

3. Epilepsy = 62

4. Mental retardation with epilepsy = 63

5. Mental retardation with behavior problems = 20

6. Substance abuse = 40

A visit to Thiruporur in 2005 showed that the resources/services created were no longer in existence and some of the old cases followed up had discontinued treatment within a few months of our exit.

We believed that it was important for us to identify the reasons for the discontinuation of treatment as well as the non-sustenance of the services created by the earlier program in order to gain new and insightful information. This in turn would help in the formulation and strategizing of similar future endeavors.

### Objectives

1. To determine the current treatment and clinical status of the patients.

2. To assess the current level of functioning and utility of the components initiated under the previous programme such as the training of the Primary Health Center (PHC) personnel, the referral system, the Citizen's group (CG) and self-employment schemes.

3. To identify the reasons that lead either to the sustenance or failure of the above initiatives.

## Methods

### Study area

The study area was the Thiruporur Block in Tamil Nadu, South India comprising of 102 villages that had been covered by the community mental health clinic from 1989 to 1999.

### Data collection

A modified version of the Psychiatric and Personal History Schedule (PPHS) [6] was used to collect information regarding the patients' present treatment status and reasons for discontinuing treatment. The PPHS was shortened to make it suitable for the field investigators to use on a rural population. The psychiatrist (RP) ascertained the clinical status using the Positive and Negative Symptom Scale (PANSS) [7], having been trained in the use of the instrument.

Supplementary qualitative information was collected using semistructured interviews about the current status of the schemes that were initiated under the previous program, their utilization and reasons for the continuation or discontinuation of the same. The qualitative information was thematically coded and classified. All Interviews were conducted either with the patient or with a family member or both. The questionnaires were first drafted in English then subsequently translated into the local language Tamil.

### Recruitment and training of staff

A social worker with training in psychiatry and community development was recruited as the coordinator of the project along with three Community Level Workers (CLWs) who had worked with us in the previous program. The CLWs were female high school graduates and lived in villages in the study area. Their pre-established bonds and their rapport with the local community greatly aided the programme. A psychiatrist was also deputed for part-time duty to the study.

The recruited staffs were briefed about the project and its objectives and over a one month period were trained in the administration of the questionnaire. Inter-rater reliability of the raters was established and was checked at regular intervals. The project coordinator also monitored the interviews and verified the data collected on a periodic basis in the field.

### Sample

For the purpose of this study, 185 patients, with diagnosis of chronic psychoses, were followed up. All of them were from the surrounding 102 villages covered by the community clinic. Ninety two interviews could be completed during which either family member or the patients themselves provided the information.

## Results

### Present status of subjects followed up

Of the 185 patients followed up only 28 (15%) had continued treatment at centers that we had referred them to at the time of our exit in 1999. More than a fifth of the patients (38 patients i.e., 21%) had died. Twenty three patients (12%) had wandered away from home and their families had no clue as to their present whereabouts. Thirty-two patients (17%) as well as their families were untraceable, as they had left the study area. The remaining 64 patients (35%) had stopped treatment after the closure of the Thiruporur clinic.

#### Deaths

**Figure 1:**
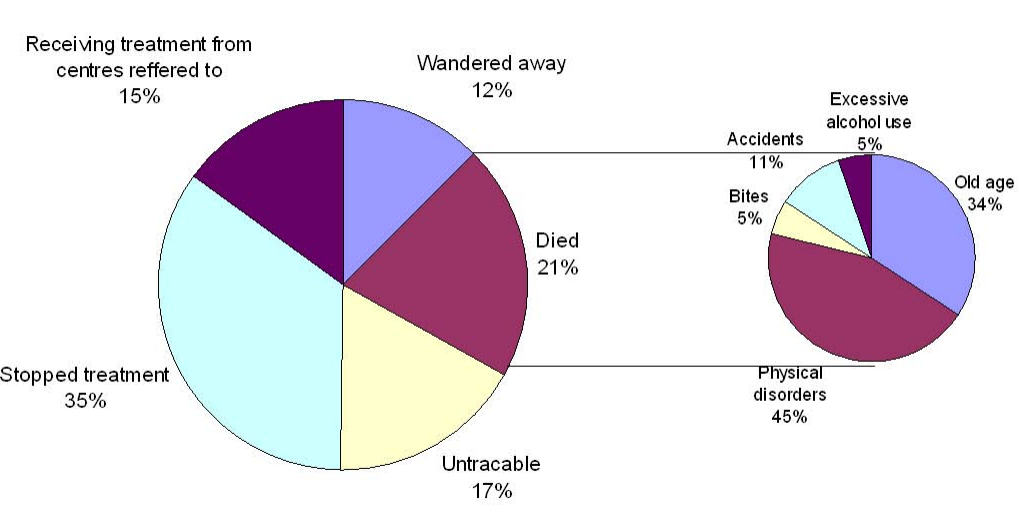
Present status of the chronically mentally ill patients registered at the Thiruporur clinic.

It was not possible to obtain very accurate information on deaths and over 90% had died at home. When the research staff visited the homes, they were informed that 13/38 had died of "old age". They had just become withdrawn, stopped eating and were never given any medical help. In the absence of any medical records for most only approximate causes of death – as indicated by events preceding death – could be ascertained (Figure 1). Seventeen (45% of deaths) of the patients died due to various physical disorders, such as paralysis, seizures, jaundice, heart attack, diarrhea, unspecified fevers. Two (5%) had died after snake and dog bite, another two (5%) had died after excessive use of alcohol, and four (11%) had died from accidents such as road traffic accidents, drowning and fire. No suicides were reported.

Patients were classified as being either symptomatic or asymptomatic based on the PANSS. Of the 64 patients who had remained untreated 16 (25%) were asymptomatic and well functioning while the remaining 48 (75%) were acutely ill and in need of treatment.

### Present status of the psychiatric consultations offered at the PHC whose personnel were trained in mental health

When the current functioning of the PHC was assessed, it was found that not a single mentally ill person had been treated there in the last five years. In fact psychiatric medication had been disbursed for less than a year after our exit.

The reasons identified for the termination of mental heath services provided by the PHC are that

1. The physician who had received training in psychiatric management had moved out and the physicians who subsequently replaced him were not trained or inclined to treat mental disorders. They considered it as an additional burden.

2. Frequent turnover of staff, especially physicians, at the PHC who often did not stay at the center for more than six to eight months.

3. Another factor that was seen to contribute to the cessation of psychiatric treatment at the PHC was their limited financial budget-psychiatric medication seemed to grab too large a piece of the pie.

### Present status of the referral system that had been established

Of the 92 patients/families who could be interviewed, only 28 patients were on regular treatment. The factors that contributed to the continuation of treatment were:

1. Proximity to the treatment facility.

2. Most of these patients were employed and found that discontinuation of treatment led to poor functioning at work which they could ill afford.

3. The family support was good-the primary care givers in most cases ensured continuity of treatment.

The reasons identified as contributing to the discontinuation of treatment were multiple and overlapping in nature. See Table 1 for reasons, frequency of responses and ranking.

**Table 1:** Reasons for discontinuing treatment

**Rank**	**Reason for discontinuing treatment**	**Frequency of ** **response (%)**
1	Distance from the referral center	92 (100%)
2	The cost of travelling to the referral center	80 (86.96%)
3	Loss of time and income of accompanying person	71 (77.17%)
4	The patient was "doing well"	23 (25%)
5	Patient continued being "ill" ever after being on treatment	19 (20.65%)

### The present status of the Citizen's Group that had been formed in the village

The CG had functioned for a year after it's inception during which general health camps were run and other activities such as organizing blood donation camps etc. The reasons identified as contributing towards the demise of the CG were

1. Lack of availability of the resource person (coordinator). The local population did not have the expertise to carry on the activities by themselves without external inputs.

2. A local parish priest who had also provided support was transferred and the subsequent replacement was not interested in mental health.

3. Money that had been collected as a corpus fund – by pooling together financial resources from the community-was exhausted and a new source could not be tapped.

4. The CG comprised of members from the general community as well as patient families as such had conflicting priorities, with more people feeling there was a greater need for general heath services rather than for mental health.

5. The patients in the area were geographically scattered over 102 villages and as such only about 10 patient/families were participating in the group regularly.

### The present status of the self-employment schemes that had been initiated

The reasons given for the non-continuation of the petty shop and goat rearing schemes were (Table 2):

**Table 2:** The present status of the self-employment schemes that had been initiated

	**Scheme**	**No. of ** **Beneficiaries**	**Duration of ** **functioning**	**Present Status**
1	Petty shop	1	6 months	Not functioning
2	Goat rearing	6	6 months to 3 years	Not functioning
3	Chicken rearing	24	7 years	Functioning

1. When the beneficiaries discontinued treatment, they relapsed and were unable to carry on with their activities.

2. Managing the petty shop and goat rearing required more systematic work than chicken rearing and when the patient fell ill, no family member was willing to take up the additional responsibility as they were busy with their own work.

3. The extreme poverty in the region that forced them to sell their herd when in dire need of money.

4. Monetary investment required to restart the activity was beyond the means of the families and the patients.

In contrast it was found that those who had been rearing chickens had been able to sustain their activity in spite of the fact that the beneficiaries under the scheme also had discontinued medication. The success of this scheme can be attributed to the following:

1. Chicken rearing was a relatively simple activity.

2. When the patient had his bad days, a family member was able to help out since the activity was not very demanding.

3. Further financial investment required to sustain the activity was negligible.

## Discussion

While in operation, the feasibility of conducting the CBR program at Thiruporur was well established [5]. The program addressed most objectives set out by the National Mental Health Program. Strategies implemented included training lay community health workers, clinical services, networking, public awareness campaigns and implementation of simple psychosocial rehabilitation strategies through lay community workers. Training of health care personnel at the primary health care facilities aimed to meet the NMHP objective of integrating mental health care with primary care [8,9].

When we made our exit from the project area in 1999, we were quite satisfied with the spectrum of activities we had initiated. However, a visit in 2005 proved to be a little unsettling and it was this, which motivated us to take up this research. In Thiruporur, our training over a period of several months did not lead to sustained psychiatric services being available to the community due to a variety of reasons. The primary factor was that physicians at PHCs were often on temporary posting and were transferred within 6 months. Other reasons included the fact that there was only one physician per PHC and he often had to deal with over 100 patients per day, leaving him little time to deal with psychiatric patients. Inclination and willingness to handle mental disorders had also much to be desired [9]. PHC's were also unable to provide psychiatric medication to the patients as they had a limited budget [9]. Budget allocation works on the principle of "greatest good for the greatest number" thereby allowing psychiatric patients to fall through the cracks.

The expectation that the patients would continue treatment in the centers to which they were referred was not realised primarily due to the fact that they found it expensive to commute to the referred center. This was despite the fact that they realized that regular treatment was necessary.

Another factor contributing to the discontinuation of treatment was the lack of awareness among families about the nature of the illness. When the symptoms were controlled and the patient had recovered sufficiently to become functional, he was seen as being "cured" and no longer in need of treatment.

Dropping out of psychiatric care is not an uncommon phenomenon, especially in the case of chronic mental illnesses. Several studies including our own have addressed this issue. While some have reported more drop-outs in improved patients who were satisfied with the level of care [10], the contradictory finding of persons dropping out because of lack of improvement was seen in other studies [11]. Our own followup study revealed that those patients who had remitted had dropped out [12].

Alternatively when a patient on treatment relapsed the family lost faith in treatment. Conflicting explanatory models about mental illness [13] and the availability of religious "healing" centers in the vicinity further compounded this problem. Our own research revealed that people held more than one explanation for a particular condition that led them to seek simultaneously different kinds of interventions. This is best exemplified by the fact that we were able to convince some of the persons with psychoses who " were residing in temples" for cure of their disorder to start taking medicines.

Regular psycho-education programs must be conducted in the community to educate the families about the nature of their relative's illness and the need for sustained medical treatment.

Most support groups for persons with mental illness are located in urban areas and comprise of families of persons with mental illness. The Citizens' group initiative of this programme was quite different, being rural and comprising of persons who had not been directly affected by mental illness. The primary objective of this group was to mobilise resources within the community that would facilitate the implementation of the CBR program. During the period of one year that the group was functional, it acted as a vital resource in the community, assisting in identifying and referring patients, organising camps, disseminating information about camp calendars and helping transportation of patients from distant villages. This group however, stopped functioning as soon as the project co-ordinator left the area. Lack of adequate leadership among the members, limited access to a mental health facility and inability to raise funds accounted for the termination of this group.

Some of these factors apply also to the continuation of the self employment schemes. The family must be co-opted in the treatment and in the self-employment schemes if it is to be sustained as evidenced by the fact that when patients relapsed they had to give up their income generating resource. Schemes that tap the innate skills that the patient and the family already possess will ensure better sustainability.

A revolving fund will provide the seed money to help start self-employment schemes. Patients/families who take a loan from the fund to start a venture will pay back in full within a predetermined timeframe, enabling the cycle to continue. These issues need to be addressed in a comprehensive manner in all future endeavors.

## Conclusion

It is eminently feasible to start a wide spectrum of mental health and related social and economic activities in a rural area. However, such initiatives however well intentioned will not be sustainable unless the family and the community are involved with the programme right from its inception. Responsible persons in the local community have to be identified to act as nodal persons for these activities. It may be prudent to entrust this responsibility to a family member, whose interest may be more tenacious. A definite link with a medical service providing mental health care also seems critical.

## Competing interests

The authors declare that they have no competing interests.

## Authors' contributions

RP and JRA carried out the study, the collection of data and were responsible for study coordination. SJ participated in its design, analysed the data and helped to draft the manuscript. RT conceived the study, participated in the design of the study and in drafting the manuscript. All the authors have read and approved the final version of the manuscript.
